# “Nonmedical” prescription opioid use in North America: a call for priority action

**DOI:** 10.1186/1747-597X-8-39

**Published:** 2013-12-01

**Authors:** Pauline Voon, Thomas Kerr

**Affiliations:** 1 British Columbia Centre for Excellence in HIV/AIDS, St. Paul’s Hospital, 608-1081 Burrard Street, V6Z 1Y6 Vancouver, BC, Canada; 2 Department of Medicine, University of British Columbia, St. Paul’s Hospital, 608-1081 Burrard Street, V6Z 1Y6 Vancouver, BC, Canada

**Keywords:** Nonmedical prescription opioid use, NMPOU, NAPOU, Not-as-prescribed opioid use, Prescription opioid misuse, Prescription drug abuse, Diversion, Pain, Substance abuse, Addiction

## Abstract

Nearly four years after the United States Congress heralded a “decade of pain control and research”, chronic pain remains a mounting public health concern worldwide. The escalating prevalence of chronic pain in recent years has been paralleled by a rise in prescription opioid availability, misuse, and associated human and social costs. However, national monitoring surveys in the U.S. and Canada currently fail to differentiate between prescription opioid misuse for the purposes of euphoria versus pain or withdrawal management. Furthermore, there is a lack of evidence-based guidelines for pain management among high-risk individuals, and a glaring lack of education for practitioners in the areas of pain and addiction medicine. Herein we propose multiple avenues for intervention and research in order to mitigate the individual, social and structural problems related to undertreated pain and prescription opioid misuse.

## Background

Nearly four years after the U.S. Congress heralded a “decade of pain control and research” (for the period of 2001 to 2010) [1-3], chronic pain management remains a mounting public health concern worldwide. Globally, over 1.5 billion people suffer from chronic pain [4]. In the U.S., pain is the most common reason for seeking medical care [5-7], and the 100 million Americans suffering from chronic pain outweighs the number of Americans with diabetes, heart disease, stroke and cancer combined [8-11]. In Canada, the estimated prevalence of chronic pain is between 15-29% [12-14]. Consequently, the cost of pain due to lost productivity and health care costs is estimated to range at least $560-635 billion USD annually [8].

The escalating problem of chronic pain has been paralleled by a distinct rise in prescription drug misuse particularly in North America [15], with a 140.5% increase in reported prescription drug misuse among the U.S. population from 7.8 million in 1992 to 15.1 million in 2003. This represents approximately 6% of the U.S. population, which exceeds the combined number of people in the U.S. who use cocaine, hallucinogens, inhalants, and heroin combined [16]. Canadian data, which have only recently been collected at a national level, estimate that approximately 4.8% of the general population used prescription opioids non-medically in 2009 [17,18]. As the demand for prescription opioids (POs) has risen, so has the availability of diverted POs and the prevalence of morbidities and mortalities associated with opioid use [19].

Importantly, of the 4.8% of the Canadian population that reported nonmedical PO use, only 2.3% (0.4% of the Canadian population) reported using POs “to get high” [17]. Thus, the remaining majority of nonmedical PO use can be attributed to factors that have been underexplored [16]. One such factor that may be fuelling the increasing demand for and availability of diverted POs is undertreated pain. One recent systematic review and meta-analysis found a 48% pooled prevalence of pain among PO misusers [20], and several studies have demonstrated a positive association between chronic pain and non-medical PO use [21,22], particularly among individuals with a history of substance misuse [23,24] who are significantly more likely to receive inadequate pain management within clinical settings [25,26]. Distinctions between PO use for euphoria versus pain or withdrawal management must be further investigated and appropriately addressed [27], since the latter may be effectively managed with medical treatment regimes (e.g., opioid agonist therapies, directly observed treatment) that may allay PO misuse and diversion. Herein, we outline several priority recommendations that may serve to mitigate the growing health and social costs of prescription opioid misuse.

## Main text

### Re-defining “nonmedical prescription opioid use”

The U.S. National Survey on Drug Use and Health (NSDUH) defines nonmedical PO use (NMPOU) as “use without a prescription of the individual’s own or simply for the experience or feeling the drugs cause”, while the Canadian Alcohol and Drug Use Monitoring Survey (CADUMS) defines NMPOU as past-year PO use “on at least one occasion to get high [or] obtained from a prescription written for someone else, bought from someone else, or obtained from any other source” [28,29]. Alternatively, the U.S. National Epidemiologic Survey on Alcohol and Related Conditions (NESARC) defines NMPOU as use “to feel more alert, to relax or quiet nerves, to feel better, to enjoy [oneself], to get high or just to see how [POs] would work” [30]. These varying and complex definitions “rely on a mix of objective and subjective measures that are difficult to verify” [29], such as subjective PO use measures including “to feel better” (NESARC) or “simply for the experience or feeling the drugs cause” (NSDUH) combined with objective PO use measures such as the individual’s source of POs [28,30]. These definitions are also problematic because they aggregate motives for and means of possessing POs into one definition and assume that PO use is a largely “nonmedical” issue despite the small proportion of PO use for euphoria [17], while the majority of PO use may be the result of medical issues such as undertreated pain or withdrawal. Thus, current definitions of NMPOU may lead to inaccurate data collection, interpretation, and counterproductive approaches such as denying POs to those with undertreated pain or withdrawal.

An improved definition may be derived from the U.S. Monitoring the Future (MTF) survey, which simply defines NMPOU as PO use “without a doctor’s orders during the past 12 months” and later differentiates various motives for PO use in the survey instrument [31]. However, this definition may not capture those who have a legitimate prescription but may take their medications not as indicated (e.g., increased dose or frequency, or alternate route or indication for administration). Therefore, we suggest the term “not-as-prescribed opioid use” (NAPOU), which recognizes that opiate use may not be “nonmedical” in nature, and includes opioid use not as indicated for the individual whether by use of someone else’s prescription or use of one’s own prescription outside of prescribed parameters (Table 1). Within this broader definition, in-depth data collection should be undertaken to dichotomize the various motives for (e.g., euphoria versus pain versus withdrawal) and means of PO use (e.g., diverted medication from street-based markets, use of another’s prescription, use of one’s own prescription outside of prescribed parameters).

**Table 1:** **Suggested definition to replace** “**nonmedical prescription opioid use**”

**Suggested definition**	**Description**
**Not-as-prescribed opioid use (NAPOU)**	The authors suggest the term **not-as-prescribed opioid use (NAPOU)**, which recognizes that opioid use may not be “nonmedical” in nature (e.g., undertreated pain or withdrawal), and includes opioid use not as indicated for the individual whether by the use of someone else’s prescription or the use of one’s own prescription outside of prescribed parameters (e.g., alternate dose, frequency, route, or indication).

### Developing evidence-based guidelines for pain management among high-risk individuals

Despite the high prevalence of pain among individuals with substance use disorders and psychosocial comorbidities [32,33], there is a severe lack of evidence to inform clinical guidelines for pain management among these complex populations. For instance, the American Pain Society’s guidelines for chronic pain management explicitly state that their recommendations for high-risk individuals are based on “low-quality evidence” and “anecdotal experience” [34]. While these guidelines reflect the state of evidence at the time (2009), there remains a paucity of high-quality research on effective pain management approaches among substance-using populations. This is reflected by the Cochrane Collaboration’s review on long-term opioid management for chronic non-cancer pain, in which the majority of studies reviewed excluded participants with a history of substance use [35]. Therefore, high-quality research on pain management for individuals with a history of substance use is urgently needed to inform evidence-based clinical practice guidelines.

### Educating practitioners in pain and addiction medicine

A U.S.-wide audit found that 40% of physicians and 48% of pharmacists received formal training in identifying prescription drug abuse, yet 74% of physicians and 83% of pharmacists refused to prescribe or dispense a controlled drug due to concerns regarding addiction, diversion or misuse [16]. Deficiencies in practitioner training in pain and addiction medicine likely contribute to inappropriate pain management and a growing opioid misuse epidemic [36]. For example, a lack of clinician training to inform patients about safe handling of POs may be contributing to the majority of adolescents (74%) with unsupervised access to prescription medications [37]. Furthermore, a recent policy by the U.S. Food and Drug Administration has required manufacturing companies to develop Risk Evaluation and Mitigation Strategies that involve providing education to patients and providers on the safe use and prescribing of extended-release and long-acting opioids, but this policy has not been effectively translated into clinical practice [38]. Therefore, pain and addiction training should be included in core medical school and residency curricula, and pain and addiction specialists should be formally recognized and incorporated into acute and community-based health care settings [36].

## Conclusions

Prescription opioid misuse remains a growing public health concern for which urgent action is required to re-define the problem at hand, develop evidence-based guidelines, and scale up education for practitioners in pain and addiction medicine. Further investigation into the role of undertreated pain as a contributor to prescription opioid misuse affords considerable opportunity to reduce personal suffering and healthcare costs. There are multiple avenues for intervention and research, and if acted upon, much of the individual, social and structural problems related to undertreated pain and prescription opioid misuse could be meaningfully addressed.

## Abbreviations

CADUMS: Canadian Alcohol and Drug Use Monitoring Survey; PO: Prescription opioid; MTF: Monitoring the Future; NAPOU: Not-as-prescribed opioid use; NESARC: U.S. National Epidemiologic Survey on Alcohol and Related Conditions; NMPOU: Nonmedical prescription opioid use; NSDUH: U.S. National Survey on Drug Use and Health.

## Competing interests

The authors declare that they have no competing interests.

## Authors’ contributions

PV and TK conceptualized the commentary and drafted the manuscript together. Both authors read and approved the final manuscript.
